# Case Report: Full-house renal-limited lupus-like nephritis in pregnancy

**DOI:** 10.3389/fneph.2025.1593927

**Published:** 2025-06-30

**Authors:** Lucille Jane Wilkinson, Sally Stauder, Brady Culpepper, Jalal Ibrahim, Vivekanand Pantangi, Prathap Kumar Simhadri

**Affiliations:** ^1^ College of Medicine, Florida State University, Tallahassee, FL, United States; ^2^ College of Osteopathic Medicine, Lake Erie College, Bradenton, FL, United States; ^3^ Department of Family Medicine, Advent Health, Daytona Beach, FL, United States; ^4^ Department of Nephrology, Advent Health, Daytona Beach, FL, United States

**Keywords:** full house nephropathy, lupus like nephritis, lupus nephropathy, renal limited lupus, nephrotic syndrome, nephrotic syndrome in pregnancy, lupus in pregnancy, membranous

## Abstract

Lupus nephropathy is a common manifestation of systemic lupus erythematosus (SLE), with immune-mediated inflammatory damage to the glomerulus leading to acute kidney injury, chronic kidney disease, and end-stage renal disease. Occasionally, patients present with renal-limited lupus nephropathy with classic full-house staining on immunofluorescence and no signs of systemic lupus. Limited data are available on renal-limited “lupus-like nephropathy” in pregnancy. A 24-year-old G1P0 woman at 14 weeks of gestation was referred to nephrology for further evaluation of 8.4g proteinuria. She was found to be ANA negative with a decreased C1q level and a renal biopsy revealing membranous nephropathy. Immunofluorescence staining was positive for IgG, IgA, IgM, C3, and C1Q, consistent with full-house pattern. She was started on 500 mg pulse dose methylprednisolone for 3 days, which was gradually tapered to 5 mg daily, and cyclosporine 75 mg BID. She delivered a healthy baby via induction at 36 weeks. Six-month follow-up revealed 1g protein on 24-hour urine collection, normal C3/C4 levels, and no signs of SLE. This case report adds to the literature discussing renal-limited “lupus-like nephropathy” in pregnancy and helps guide further management of this condition.

## Introduction

Systemic lupus erythematosus (SLE) is a systemic autoimmune disease recognized by a constellation of clinical, laboratory, and histopathological findings (1). Lupus nephropathy is a common manifestation of SLE, with immune-mediated inflammatory damage to the glomerulus leading to acute kidney injury (AKI), chronic kidney disease (CKD), and end-stage renal disease. The literature has described a small subset of patients demonstrating renal-limited “lupus-like nephropathy” in pregnancy, defined by pathological biopsy findings typical of lupus nephropathy without systemic manifestations or laboratory biomarkers of the disease (2). A full-house stain, identified by positive immunofluorescence staining of all three isotypes of immunoglobulins, is a pathological finding highly associated with SLE (3). However, few studies have identified renal-limited “lupus-like nephropathy” in pregnancy (4).

## Case report

A 24-year-old G1P0 woman at 14 weeks gestation was referred to nephrology for evaluation of proteinuria after 24-hour urine collection revealed 8.4 grams of proteinuria. At presentation, she complained of bilateral lower extremity edema for over two weeks. She had no significant past medical conditions or family history of kidney disease or autoimmune disease. She denied any fevers, rashes, joint pain, orthopnea, paroxysmal nocturnal dyspnea, or use of NSAIDs, alcohol, tobacco products, or use of nephrotoxic agents. Physical exam findings were significant for +1 bilateral pitting lower extremity edema.

**Figure 1:**
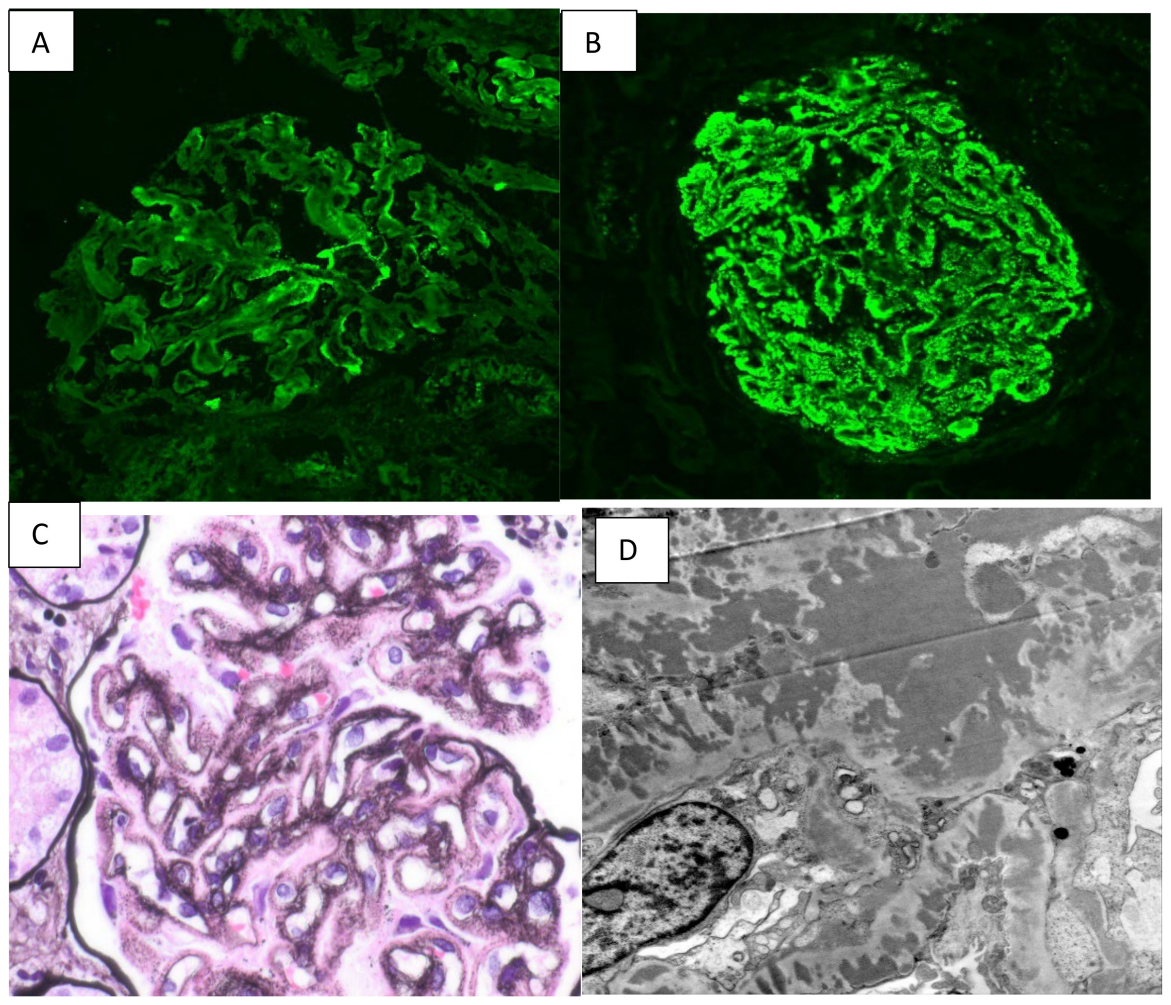
**(A)** shows 1+ C1q staining. **(B)** shows 3+ Ig G staining on immunofluorescence. **(C)** shows diffuse thickening of capillary walls with fine lucencies on silver stain. **(D)** shows the presence of global subepithelial and intramembranous electron-dense deposits.

At 16 weeks gestation, 24-hour urine revealed 14 grams of proteinuria and a serum albumin level of 1.5 gr/dL. She was hospitalized for the management of nephrotic syndrome and was found to be ANA negative with a decreased C1q complement of 94 (102–171 mg/L), but was negative for other SLE autoantibodies (anti-Smith and anti-dsDNA) and had normal C3 (101 mg/dL, reference range 90–180 mg/dl) and C4 complement (33 mg/dl, reference range 10–40 mg/dL). She tested negative for lupus anticoagulant, cryoglobulins, paraproteinemia, and viral hepatitis. She underwent an ultrasound-guided kidney biopsy, which revealed membranous nephropathy with positive staining for IgG, IgA, IgM, C3, and C1Q, consistent with full-house immunoglobulin staining as shown in **Figure 1**.

**Figure 2:**
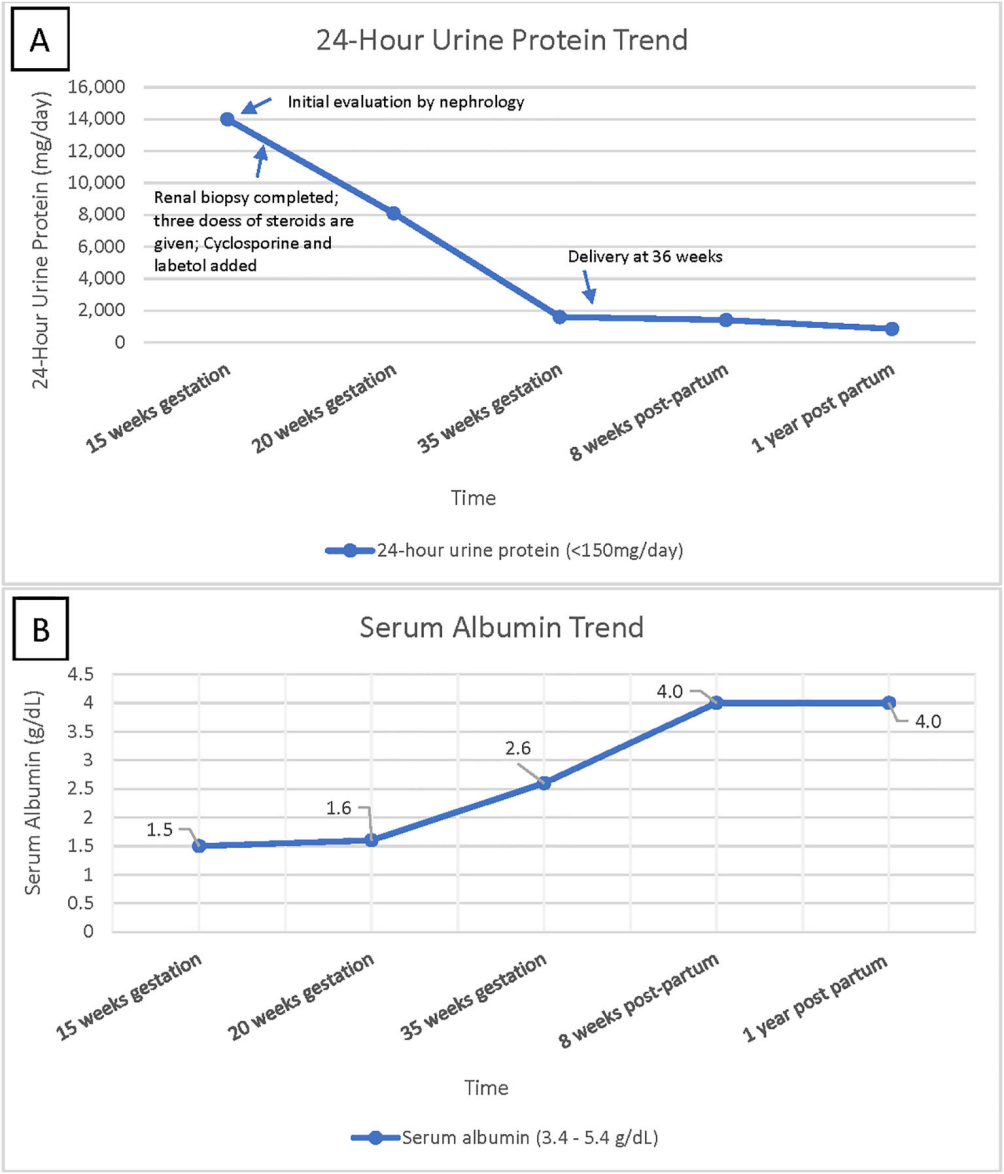
**(A)** shows 24-hour urine protein trend over time, highlighting improvement in urine protein leakage over time. **(B)** shows serum albumin trend over time, indicating patient’s return to baseline albumin level after treatment.

She was started on cyclosporine 75 mg BID and 500 mg pulse dose methylprednisolone for 3 days, which was then continued at 10 mg daily. She was maintained on therapeutic enoxaparin and aspirin 81 mg daily during the pregnancy to prevent thrombotic complications. Proteinuria started improving with the above regimen; it reached < 2gr/day and the serum albumin improved to 2.6 by 35 weeks of gestation, as depicted in **Figure 2**. She was continued on this medication regimen until induction at 36 weeks, when she delivered a healthy baby with 50% percentile birth weight. A 10-week post-partum follow-up revealed 1 gram of protein on a 24-hour urine collection and normal C3 and C4 levels. She continued to exhibit no signs of SLE, and the C1q complement level normalized. Methylprednisolone and cyclosporine were discontinued, and dapagliflozin and losartan were initiated for blood pressure management and renal protection. Her angiotensin receptor blocker (ARB) dose was maximized, and at one-year post-partum she is doing well with proteinuria improved to < 500 mg/day.

## Discussion

A 24-year-old pregnant female presented with pathological evidence of renal limited “lupus-like nephropathy” without clinical or laboratory evidence consistent with SLE. This case adds to the literature describing this condition. More evidence is essential to guide proper treatment and management of cases like this in the future.

In congruence with the small number of reported cases of renal limited “lupus like nephropathy”, our patient presented during pregnancy with edema and no history of kidney disease or SLE. Compared to other cases, our patient had significant proteinuria of 14 grams, whereas other patients ranged from 3 grams to 8 grams with the exception of one case of 16.6 grams of proteinuria as shown in **Table 1** (4, 5). Of the few studies reporting on renal limited “lupus-like nephropathy” in pregnancy, only the adolescent patients delivered small for gestational age-sized babies, while the other patients, including our patient, delivered normal for gestational age-sized babies (4, 5). Only two patients have been reported in the literature to develop SLE after complete remission of lupus-like nephropathy during pregnancy, one at 7 years and the other at 11 years after the pregnancy as shown in **Table 1**.

**Table 1:** Comparison to other reported cases.

Reference	Patient age	Gestational age	Proteinuria at referral (g/dL)	Treatment	Pregnancy outcome (birth weight percentile)	Renal outcome
Membranous full-house pattern						
Present case report	24	14	8.4	Methylprednisolone, cyclosporine	Delivery at 36 weeks (50^th^)	Complete remission
Orozco-Guillén AO, et al. (4)	23	22 weeks	5.65	Azathioprine, prednisone	Delivery at 34 weeks (58^th^)	SLE diagnosis after 7 years
Orozco-Guillén AO, et al. (4)	16	26 weeks	8.0	Azathioprine, prednisone	Delivery at 38 weeks (3^rd^)	Complete remission
Orozco-Guillén AO, et al. (4)	14	21 weeks	16.6	Cyclophosphamide, methylprednisolone, prednisone	Delivery at 36 weeks (1^st^)	Complete remission
Orozco-Guillén AO, et al. (4)	17	30 weeks	3.2	Tacrolimus, prednisone	Delivery at 38 weeks (7^th^)	Complete remission
Yamada T, et al. (5)	34	8 weeks	3.4	Methylprednisolone, prednisolone	Delivery at 37 weeks	SLE diagnosis 11 years later
Membranoproliferative full-house pattern						
Orozco-Guillén AO, et al. (4)	32	9 weeks	3.9	Cyclophosphamide, methylprednisolone, prednisone	Molar pregnancy termination at 9 weeks	Complete Remission
Orozco-Guillén AO, et al. (4)	31	27–30 weeks	3.9	Mycophenolate mofetil, prednisone	Delivery at 32 weeks (36^th^)	Persistent proteinuria
Orozco-Guillén AO, et al. (4)	26	8 weeks	4.6	Cyclophosphamide, prednisone	Molar pregnancy termination at 8 weeks	Renal impairment, proteinuria

Similar to our case, other studies emphasize the potential for good long-term outcomes with the use of proactive, aggressive treatment of glomerulonephritis in pregnancy (4, 5). It is important to note that some patients were lost to follow-up after a certain number of years, so it is difficult to predict the possible outcomes for these patients (4).

In regard to cases of lupus-like nephritis in non-pregnant patients, prior research has indicated that 25% of these patients develop SLE after a mean follow-up period of 5 years and the renal prognosis of these patients appears to be better than that of patients with lupus membranous glomerulonephritis (2). There have been a range of studies describing cases of non-pregnant patients with lupus like-nephritis with variable treatment regimens and outcomes, ranging from complete remission to end stage renal disease (1, 2, 6). This highlights the need for continued research in this topic to elucidate patterns and the mechanism of this pathology.

## Conclusion

There is a small but growing number of cases reporting renal-limited lupus-like membranous nephropathy in pregnancy. Our case report describes a 24-year-old female presenting with renal-limited full-house membranous nephropathy at 14 weeks of gestation. The clinical course outlined in this case report adds to the limited amount of knowledge we have regarding proper treatment plans and prognoses in this disease.

### Patient perspective

Our patient expressed her heart felt gratitude for being there with her during her pregnancy journey in the management of nephrotic range proteinuria. She is happy with the progress she had with the treatment during the pregnancy and her overall outcome.

## Data Availability

The original contributions presented in the study are included in the article/**Supplementary Material**. Further inquiries can be directed to the corresponding author.
